# Structural and functional differences in gut microbiome composition in patients undergoing haemodialysis or peritoneal dialysis

**DOI:** 10.1038/s41598-017-15650-9

**Published:** 2017-11-15

**Authors:** Vanessa Stadlbauer, Angela Horvath, Werner Ribitsch, Bianca Schmerböck, Gernot Schilcher, Sandra Lemesch, Philipp Stiegler, Alexander R. Rosenkranz, Peter Fickert, Bettina Leber

**Affiliations:** 1 Division of Gastroenterology and Hepatology, Department of Internal Medicine, Medical University of Graz, Graz, Austria; 2 Division of Transplantation Surgery, Department of Surgery, Medical University of Graz, Graz, Austria; 3 Clinical Division of Nephrology, Department of Internal Medicine, Medical University of Graz, Graz, Austria; 4 Intensive Care Unit, Department of Internal Medicine, Medical University of Graz, Graz, Austria; 5 Center of Biomarker Research in Medicine (CBmed), Graz, Austria

**Keywords:** Microbiome, End-stage renal disease

## Abstract

Complications of end-stage renal disease (ESRD) are critically related to inflammation. The gut microbiome is a key driver of inflammation. Since dialysis modalities may differently influence the gut microbiome, we aimed to compare the effects of haemodialysis (HD) and peritoneal dialysis (PD) on patients’ gut microbiome composition and function. We therefore studied faecal microbiome composition and function as well as inflammation and gut permeability in 30 patients with ESRD (15 HD, 15 PD) and compared to 21 healthy controls. We found an increase in potentially pathogenic species and a decrease in beneficial species in patients on HD and to a lesser extend in patients on PD when compared to controls. These changes in taxonomic composition also resulted in differences in predicted metagenome functions of the faecal microbiome. In HD but not in PD, changes in microbiome composition were associated with an increase in c-reactive protein (CRP) but not with intestinal inflammation or gut permeability. In conclusion microbiome composition in ESRD differs from healthy controls but also between modes of dialysis. These differences are associated with systemic inflammation and cannot completely be explained by dialysis vintage. The mode of renal replacement therapy seems to be an important driver of dysbiosis in ESRD.

## Introduction

Chronic kidney disease (CKD) is a major health burden since 15% of the adult population and up to 25% in high risk groups such as diabetes have CKD^1,2^. Despite renal replacement therapy in the western world, mortality of patients with end stage renal disease (ESRD) remains high; mainly due to cardiovascular disease as well as bacterial infections and sepsis^3–5^, Low grade inflammation could be a plausible link between infections and increased risk of cardiovascular morbidity^6,7^. One of the key drivers of inflammation are translocated bacterial products from the intestinal tract, highlighting the importance of the intestinal microbiome in morbidity and mortality of patients with ESRD^8^. The gastrointestinal tract has also been recognized as a source of uremic toxin generation which further contributes to chronic inflammation^9,10^. Serum endotoxin levels as a marker of bacterial translocation is an independent predictor for mortality in ESRD^11,12^. Therefore, detailed studies of gut microbiome composition in ESRD are warranted.

The first systematic analysis of the intestinal microbiome in 24 ESRD patients by phylogenetic microarray analysis revealed several abnormalities in the composition of the intestinal microbiome: 190 OTUs from Brachybacterium, Catenibacterium, Enterobacteriaceae, Halomonadaceae, Moraxellaceae, Nesterenkonia, Polyangiaceae, Pseudomonadaceae, and Thiothrix families were increased in ESRD patients and from animal experiments the authors concluded that uraemia was the main driver for dysbiosis^13^. Further analysis of these data revealed that ESRD patients exhibit an increase of bacterial families possessing urease, uricase, and indole and p-cresol forming enzymes, whereas families with butyrate-forming enzymes were decreased^14^. In a recent study, microbial metabolites have been found to be independent predictors of mortality and cardiovascular disease^15^. Besides uraemia, several factors such as typical dietary restrictions in chronic kidney disease, co-medication and comorbidities can contribute to dysbiosis^16^. A longitudinal study in kidney transplant recipients showed that transplantation and the concomitant change in medication causes a rapid improvement in the intestinal microbiome composition^17^. However, it remains enigmatic whether the type of renal replacement therapy has any impact on intestinal dysbiosis. Recently, PD was associated with stronger changes in diversity and microbiome composition compared to HD in paediatric patients, however, no correlation between diversity and inflammation was observed^18^. We recently showed that in adults, dialysis modality and not renal function *per se* determines the development of innate immune dysfunction and endotoxemia in adult CKD-patients. Recently it has been shown that patients undergoing HD have a 2.4 fold higher risk of death compared to PD patients in a large propensity matched study, that could not be attributed to initial use of central venous catheters or lack of pre-dialysis care^19^. HD patients have also been shown particularly prone to endotoxemia and subsequent innate immune dysfunction and innate immune function seems to improve after kidney transplantation^20^. These findings raise the hypothesis that the gut microbiome as major contributor to bacterial translocation and inflammation might be involved in these differences.

To determine the specific effects of HD and PD we therefore analysed the composition and the predicted function of the faecal microbiome from adult HD and PD patients and relate it to clinical data, co-medication, comorbidities, inflammation and gut permeability since this may give pivotal information for optimized patient care.

## Results

Fifty-one faecal samples from 21 healthy controls and 30 ESRD-patients were assessed. Of the 30 patients with ESRD, 15 underwent HD and 15 PD. HD and PD patients showed comparable characteristics concerning age, gender, comorbidities, underlying kidney disease, and co-medication. Dialysis vintage was significantly shorter in PD patients. Controls were comparable to both patient groups regarding age, gender and BMI. Patient characteristics are shown in Table 1. Dialysis vintage showed a significant positive correlation with age and a weaker but still significant negative correlation with BMI (r = 0.449, p = 0.002; r = −0.296, p = 0.030).

**Table 1:** Baseline characteristics and biochemistry.

	HD (n = 15)	PD (n = 15)	HD versus PD	Control (n = 21)	Control versus HD/PD
General characteristics					
gender (f/m)	5/10	3/12	ns	12/9	ns
age (years)	61 (54;71)	62 (54/69)	ns	58 (53/62)	ns
Charlson comorbidity score	6 (5;8)	6 (5;8)	ns	—	ns
BMI (kg/m2)	23,7 (21,5; 30,8)	28,7 (23,9; 32,6)	ns	25,3 (23,4;25,8)	ns
Dialysis vintage (months)	70 (40; 197)	25 (15;74)	p = 0.011	—	—
diabetes mellitus (y/n)	6/9	4/11	ns	—	ns
cardiovascular complications (y/n)	7/8	8/7	ns	—	ns
vascular access (shunt/catheter)	10/5	—	—	—	—
Etiology					
immunological	5	6	ns	—	—
non-immunological	8	7	ns	—	—
other	2	2	ns	—	—
Medication					
proton pump inhibitors (y/n)	7/8	7/8	ns	2/21	ns
sevelamer (y/n)	9/6	9/6	ns	—	—
immunosuppression (y/n)	1/14	2/13	ns	—	—
Laboratory parameters					
GFR (ml/min)	6,0 (5,9; 9,3)	7,9 (7,3; 14,0)	ns	77,6 (73,4; 86,6)	p < 0,001/ p < 0,001
creatinine (mg/dL)	7,4 (4,6; 9,2)	5,9 (4,1; 7,8)	ns	0,9 (0,8; 1,1)	p < 0,001/ p < 0,001
urea (mg(dL)	89 (66; 104)	99 (89; 125)	ns	32 (25;36)	p < 0,001/ p < 0,001
phosphate (mmol/L)	1,5 (1,1;2,1)	1,3 (1,2; 1,6)	ns	1,0 (0,9; 1.0)	p < 0,001/ p = 0,002
albumin (g/dL)	3,8 (3,7;4,3)	3,8 (3,5; 4,1)	ns	4,3 (4,3; 4,4)	ns
CRP (mg/L)	4 (1,6; 12.9)	3,6 (1,4; 6,9)	ns	1,2 (0,9; 1,8)	p = 0.019/ns

### Diversity and variability

**Figure 1:**
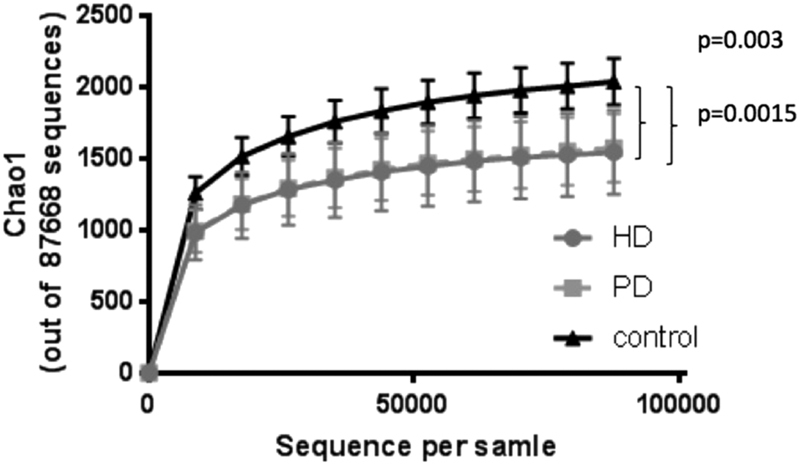
Chao1 index as a measure of species richness in patients undergoing HD or PD and in controls. Chao1 index is significantly higher in controls compared to HD (0.0015) or PD (p = 0.003). There is no difference in species richness between HD and PD.

**Figure 2:**
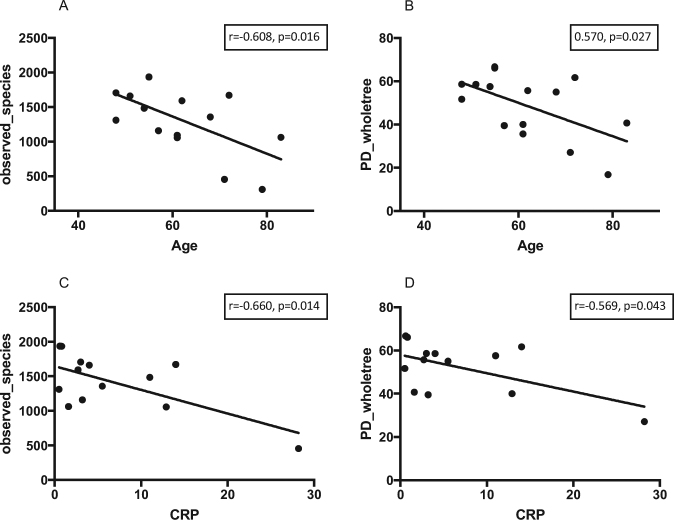
Correlation between the alpha diversity measures observed species and phylogenetic distance and age as well as CRP. Pearsons correlation coefficients are shown in the upper right corner of each diagram.

**Figure 3:**
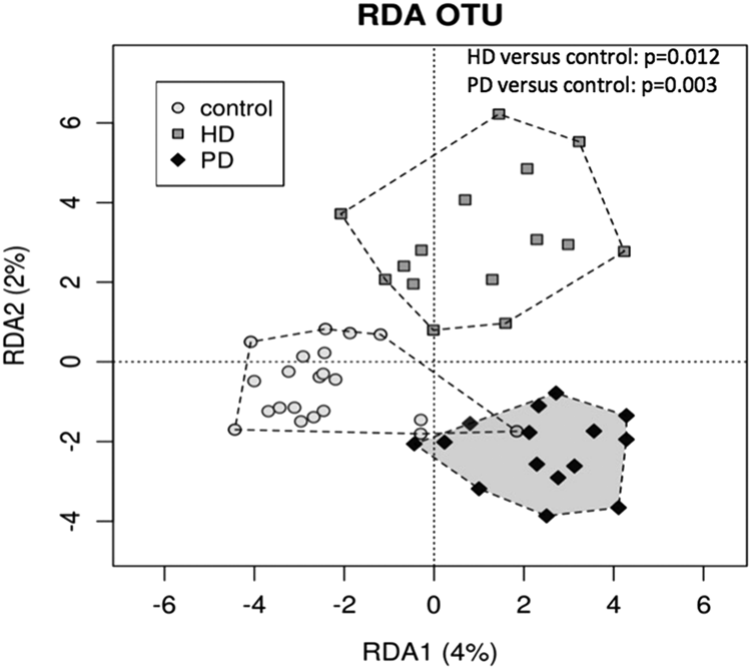
Redundancy analysis for HD patients’ microbiomes and PD patients’ microbiomes are significantly different from controls but not between each other. Bonferroni corrected p values are given in the upper right corner of the figure.

Alpha Diversity as measured by Chao1 index was significantly lower in HD and PD patients compared to controls (p < 0.05), but did not differ between HD and PD patients (Fig. 1). Similar results were found for observed species and phylogenetic diversity. Alpha-diversity did not correlate with dialysis vintage, BMI, age or CRP levels in the whole cohort. When analysing the groups separately, in HD patients, observed species and phylogenetic diversity whole tree correlated negatively with age (r = −0.608, p = 0.016; r = −0.570, p = 0.027) and CRP (r = −0.660, p = 0.014; r = −0.569, p = 0.043) (Fig. 2). Age did not correlate with CRP. No specific clustering was observed on PCoA plots based on Bray Curtis dissimilarities, accordingly dissimilarities did not differ significantly between and within all three groups. Redundancy analysis showed significant differences between controls and HD (p = 0.012) or PD (p = 0.003; see Fig. 3). HD and PD did not differ significantly on redundancy analysis. Multifactorial redundancy analysis between HD and PD patients did not show any effect of co-medication (proton pump inhibitor, immunosuppression, or phosphate binder) and comorbidities (cardiovascular or diabetes) on microbiome composition.

### Taxonomic composition

While the gut microbiome composition of HD and PD patients did not differ from controls on phylum level, the following classes were differentially abundant: Bacilli, Epsilonproteobacteria and Gammaproteobacteria were highest in HD, Alphaproteobacteria were highest in PD and Erysipelotrichia was highest in controls. On order level, this was reflected by significant changes in the abundance of Lactobacillales, Campylobacterales, Enterobacteriales, Erysipelotrichales and Gastranaerophilales. On family level Comamonadaceae, Campylobacteraceae, Streptococcaceae, Enterobacteriaceae, Bacteroidales S24-7 group, Rhodospirillaceae, Erysipelotrichaceae and two uncultured bacteria were differentially abundant. At genus level 20 genera showed a significantly different abundance. Of these genera using Linear discriminant analysis effect size (LefSe) Coprococcus, Holdemanella, Asteroleplasma, Paraprevotella, Prevotella, Romboutsia, Lachnospiracea UCG008, and Pelomonas, were attributed to controls, Thalassospira, Eisenbergiella, Ruminococcaceae, and Coprococcus were attributed to PD and Escherichia-Shigella, Streptococcus, Enterobacter, and Blautia were attributed to HD.

**Figure 4:**
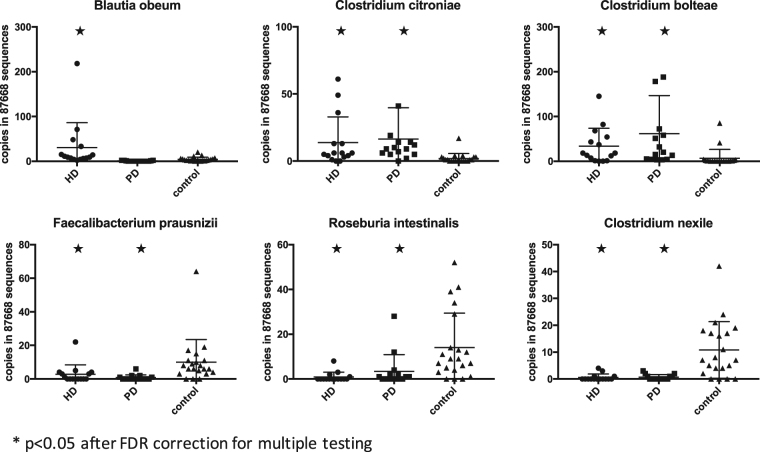
Abundance of the 6 differentially abundant OTUs *Blautia obeum*, *Clostridium citroniae*, *Clostridium bolteae*, *Faecalibacterium prausnizii*, *Roseburia intestinalis* and *Clostridium nexile* in HD, PD and controls. Stars indicate significant differences compared to controls. Data are presented as a scatter plot with mean and standard deviation. Asterisks indicate significant differences to controls (p < 0.05), p-values are FDR corrected.

On OTU level 21 OTUs were differentially abundant between the groups. In order to identify the most important species, supervised learning based on a Random Forrest Classifier was performed. Of the 21 OTUs that were significantly different on group significance analysis 8 were found among the top 10 most important OTUs to discriminate between groups. *Blautia obeum*, *Clostridium citroniae* and *Clostridium bolteae* were significantly more abundant in HD patients and the latter two also in PD patients compared to controls. *Faecalibacterium prausnizii*, *Roseburia intestinalis* and *Clostridium nexile* were significantly less abundant in HD, but not in PD patients compared to controls. (Fig. 4) Two of the 3 above mentioned OTUs identified as *Blautia obeum* were differentially abundant between HD and PD patients. Both OTUs were also identified by supervised learning as the most important discriminators between HD and PD patients.

In our cohort 7 patients died during follow up (median follow up 17 (15;29) months), all of them from the HD group. Those who died had a significantly lower alpha diversity with a significantly lower Chao1, observed species and phylogenetic diversity at the time of study. No differences in beta diversity were found between survivors and non-survivors. Also, no differentially abundant OTUs were detected between survivors and non-survivors, however the abundance of the genus Anaerococcus was significantly higher in patients who died during the observation period compared to survivors.

### Functional predictions

**Figure 5:**
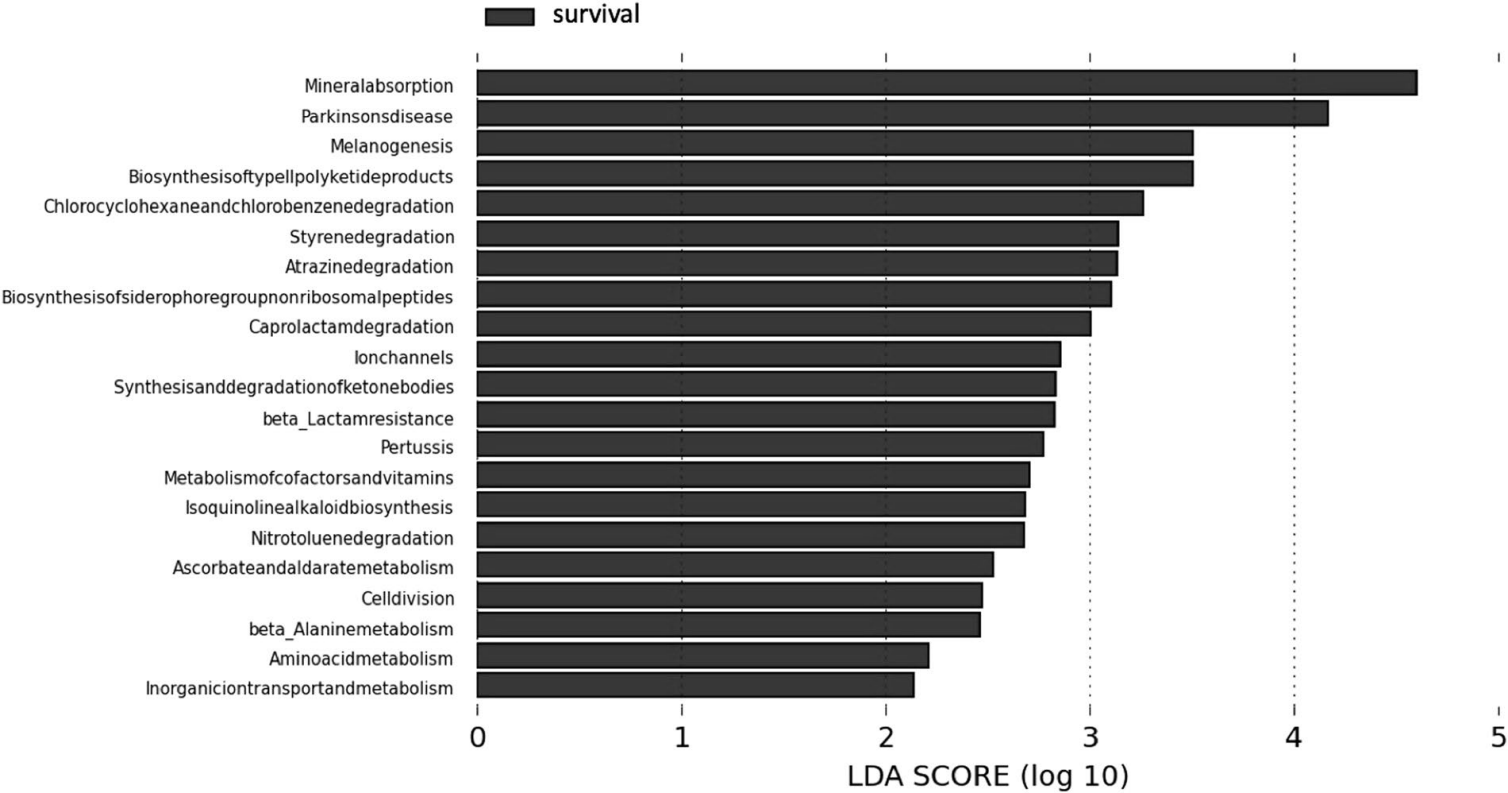
LEfSe plot showing 21 predicted metagenome pathways from PICRUSt analysis attributed to survival when comparing survivors and non-survivors in the whole cohort of HD and PD patients. None of these differentailly abundant pathways showed a statistically significant difference.

Metagenome prediction using PICRUSt showed that alpha diversity (using Chao1) was significantly lower in PD compared to HD and controls. LEfSe showed 4 significantly discriminative metagenome pathways (renin-angiotensin system, glycosphingolipid biosynthesis, isoflavonoid biosynthesis and vasopressin regulated water reabsorption) between controls and ESRD patients and 1 significantly discriminative metagenome (ion coupled transporter) pathway between HD and PD. Although the taxonomic microbiome composition was not hugely different between patients who died within the observation period compared to those who survived, 21 discriminative metagenome pathways could be found to be associated with survival when employing LEfSe analysis (Fig. 5), however, none of these pathways showed a statistically significant difference.

### Gut permeability and inflammation in ESRD

**Figure 6:**
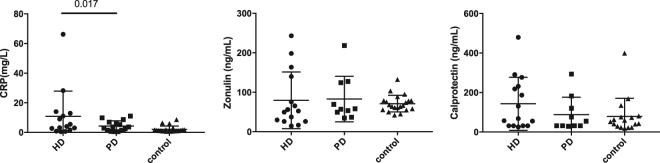
Biomarker of inflammation, gut permeability and intestinal inflammation (faecal calprotectin) in HD, PD and controls. Data are presented as a scatter plot with mean and standard deviation.

CRP as a biomarker of inflammation was significantly elevated in HD but not in PD patients compared to controls (Fig. 6). CRP correlated positively with dialysis vintage only in HD patients (r = 0.702, p = 0.007). Calprotectin as a marker of intestinal inflammation and zonulin as a marker of gut barrier integrity were not different between patient groups and controls (Fig. 6). Within the HD patient group there was a correlation between faecal calprotectin (r = 0.565, p = 0.044) and zonulin (r = 0.762, p = 0.002) and CRP levels.

## Discussion

In this cross-sectional pilot study, we demonstrate that the faecal microbiome from patients with ESRD differs from healthy controls and that the mode of renal replacement therapy influences the composition and function of the faecal microbiome. Alterations in microbiome composition are associated with elevations of inflammatory biomarkers. We have recently shown that endotoxemia and consecutively neutrophil dysfunction was most pronounced in HD patients compared to patients with CKD 3-5, PD or after kidney transplantation^20^.

In patients on renal replacement therapy the occurrence of systemic endotoxaemia and inflammation is a well-recognized phenomenon. Nowadays, where non-ultrapure dialysis water as a source can be largely excluded^21^, bacterial translocation across an impaired mucosal gut barrier is a likely source. We previously showed that increased endotoxin levels were predictive for death due to infections during follow up^20^. In our study cohort the type of vascular access in the HD group (catheter n = 4; shunt n = 11) did not impact on serum levels of endotoxin and CRP.

We also assessed gut permeability and intestinal inflammation in our study cohort by measuring zonulin and calprotectin. Both markers were not elevated in our cohort of ESRD patients, in contrast to our previous findings, where we found elevated DAO, lipopolysaccharide binding protein and sCD14 levels in chronic kidney disease stage G3-5 patients, HD and PD patients and patients after kidney transplantation^20^. Zonulin is considered to be a valid biomarker of tight junction integrity of the gut in various diseases^22,23^. In ESRD elevated serum zonulin levels have been demonstrated by others and linked to inflammation and metabolic diseases^24,25^. No data on faecal zonulin or calprotectin levels in ESRD could be found in literature. The reason for this apparent discrepancy in our study could be based on the small sample size and the high inter-individual variation of these markers in stool, since increased gut permeability and subsequent bacterial translocation is already well described especially in HD patients^26–30^.

An altered gut microbiome composition is thought to be the driving force for gut barrier disruption and inflammation^31^ and could also contribute to the increased risk of infection. As in many other acute and chronic diseases, with the emergence of modern sequencing and bioinformatics techniques, the gut microbiome has been studied in ESRD. The first study in humans showed alterations in 190 bacterial OTUs when comparing 24 ESRD patients to healthy controls. In animal experiments uraemia caused a decrease in Lactobacillaceae and Prevotellaceae families^13^. In a subsequent further analysis of these data using a targeted metagenomics approach, an increase of bacterial families possessing urease, uricase, and indole and p-cresol forming enzymes and a decrease in families with butyrate-forming enzymes was noted^14^. All patients in this study were on HD. This first study already allowed the conclusion that ESRD is associated with strong structural and functional changes in gut microbiome composition. We could confirm the finding of structural and functional alterations of the microbiome with our study. On OTU level, we found an increase in *Blautia obeum* (or *Ruminococcus obeum* as it was previously termed^32^) in HD which may be an indicator of metabolic stress^32^. On the other hand we found a decrease in *Faecalibacterium prausnizii* in HD and to a lesser extend in PD, a highly metabolic active commensal that is reduced in a variety of intestinal and extra-intestinal diseases^33^. Furthermore, we found a shift in the composition of Clostridiales. *Clostridium bolteae* and *citroneae* were more abundant in ESRD patients whereas *Clostridium nexile* was less abundant. *Clostridium bolteae* has been associated with autism and acute rejection after transplantation^34,35^ *Clostridium citroneae* has been found in isolates from clinical microbiology samples^36^. The role of *Clostridium nexile* is unclear; on the one hand, it has been associated with vegetarian diet but on the other hand it has been found in colorectal carcinoma samples^37,38^. In our study, changes were mainly detected in OTUs with relatively low abundance, however a uniform directionality could be observed in these changes. The reduction of one *Faecalibacterium prausnitzii* OTU alone might not be clinically relevant; however the simultaneous reduction of *Roseburia intestinalis* and the genus *Lachnospira* could have a cumulative effect on the bioavailability of butyrate in the intestine. Accordingly, the increase of one potential pathogen in patients on HD might be an indicator for changed growth conditions in the microbial ecosystem. The functional relevance of the observed changes can also be explained by the principle of long-tailed community structure with few predominating abundant taxa and a long tail of low abundance taxa. These low abundance taxa in particular are crucial to our understanding of microbial ecosystems, as they represent the vast functional diversity^39^.

Since the gut microbiome composition is largely influenced by diet and drugs, it is likely that these factors are important for shaping the microbiome in ESRD. Patients with ESRD have to take several drugs, which are likely to influence microbiome composition, such as phosphate binders, immunosuppression, antibiotics or proton pump inhibitors^39^. In a longitudinal study the influence of kidney transplantation, where medication of patients changes completely and overall health status improves, has been assessed. In this study blood, oral, urinary and rectal microbiome composition was analysed. Microbiome composition at all sites improved dramatically and persistently after kidney transplantation with a decrease in opportunistic pathogens and an increase in richness. However, due to the large inter-individual differences, despite a large sample size, it was not possible in this study to identify any universal diagnostic marker for the risk of complications^17^. In another pilot study studying serial faecal samples from 26 patients during the first three months after kidney transplantation, distinct alterations of the gut microbiome were associated with complications such as diarrhoea, acute rejection or urinary tract infections, also requiring changes in medication^40^. In our study we found no clear correlation between the intake of drugs known to influence the microbiome, such as proton pump inhibitors, phosphate binders or immunosuppression with changes in microbiome composition, however the design as a cross-sectional study and sample size in our groups was too small to draw firm conclusions in this regard.

In a systemic disease, it is likely that not only the gut microbiome is affected. This has already been proven for ESRD for example for the periodontal microbiome, where the microbiome in subgingival plaques of 14 ESRD patients with periodontal disease showed decreased alpha-diversity and changes in beta-diversity as well as taxonomic composition compared to controls with periodontal disease but without renal disease. In this study dialysis vintage correlated negatively with community diversity of the microbiome, suggesting that the degree of dysbiosis is related to the duration of ESRD^41^. Our study also underpins this finding since we found a negative correlation between alpha-diversity indices and age as well as CRP in HD patients and CRP was positively correlated with dialysis vintage, suggesting that long-term HD is associated with increased inflammation possibly linked to dysbiosis.

In addition to previously published data, our study adds to the understanding of the role of the gut microbiome in ESRD, because we studied HD and PD patients. We could show that the changes in microbiome composition were more pronounced in HD patients compared to PD patients. This study is to our knowledge the first study in adult ESRD patients comparing the microbiome between HD and PD patients. In children, PD was associated with more pronounced microbiome changes, such as a decreased diversity and an increase in potentially harmful taxa. Dialysis vintage was similar between HD and PD in this paediatric cohort. In this study, it was not possible to link the differences in microbiome composition to biomarkers of inflammation, bacterial translocation or uraemia^18^.

Besides dialysis vintage, which is likely to be the strongest factor in our dataset, also the dialysis procedure itself or differences in the inevitable dietary restrictions may explain the differences we found between HD and PD. HD may cause intestinal hypoperfusion due to a reduction in splanchnic blood flow in the context of compensatory mechanisms to retain hemodynamic stability during ultrafiltration. It has been hypothesized that this might cause disturbances of gut barrier integrity. We have previously shown that hepato-splanchnic blood flow substantially decreases during HD as a result of an active splanchnic vasoconstriction thus weakening the gut barrier, especially in diabetic subjects^42^. Also HD removes uremia toxins in a discontinuous way whereas PD works continuously.

Since PD patients are less prone to hyperkalemia compared to HD patients^43^, dietary recommendations differ in PD compared to HD patients^44^. Non-adherence to restrictions is linked to higher morbidity and mortality in ESRD patients and because of that restrictions are not divisible from the mode of dialysis. Therefore, the clinical relevance of an investigation of the microbiome in these patients treating dietary restrictions as confounders rather than a key element of therapy is disputable. Dietary restrictions are considered less challenging by PD patients, which also improves their quality of life^45,46^. This may lead to a more diverse diet, followed by changes in microbiome composition. Besides the therapeutic restrictions, individual dietary habits could also have minor additional influence on the gut microbiome, however since our cohort was similar in their BMI and serum albumin levels, macronutrient malnutrition does not seem to play an overt role in our patients. Furthermore, all patients were recruited from the same centre and therefore share a confined geographical region with little deviation in typical food selection, and recruitment was done in a relatively small time period, minimizing seasonal effects on diet.

Besides its role in gut barrier homeostasis and inflammation, the gut microbiome also plays an important role in the metabolism of uremic toxins^47^ and has been associated to cardiovascular risk factors such as TMAO^48^. In a study using denaturating gel electrophoreses, an alteration in the composition of the microbiome and an association with cardiovascular risk biomarkers was found^49^. Our study was not designed and powered to test uremic toxins, markers of cardiovascular risk or outcome. No relevant differences in microbiome composition and function could be found in our study regarding cardiovascular comorbidities, but the microbiome of those patients who died during follow up was slightly different in its composition and functions compared to the microbiome of survivors. This however is mainly attributable to the fact that in our study cohort only patients from the HD group died during follow up. This again encourages further, larger studies with a longitudinal design, to potentially be able to identify prognostic or therapeutic biomarkers originating from the microbiome.

The limitation of our study is the cross sectional, observational nature of the study which does not allow proving causality. Further studies are required to assess prognostic implications and potential therapeutic targets in this population. Furthermore, dietary intake was not assessed in detail this study and therefore does not allow to conclude, how nutritional differences between HD and PD group could contribute to the observed changes. Further studies are required to assess the role of nutrition, the prognostic implications and potential therapeutic targets in this population.

In summary, our study shows for the first time, taxonomic composition of the faecal microbiome in adult patients with ESRD is different in patients undergoing HD and PD, with a more pronounced dysbiosis in HD. These changes can partly but not completely be attributed to the differences in dialysis vintage between HD and PD patients in our cohort, therefore we propose, that the mode of renal replacement therapy is an important factor in the observed changes in microbiome composition and function. This may have implications on inflammation and subsequently on the development of complications and mortality in ESRD and may ultimately explain the differences in mortality between HD and PD patients. Further studies are warranted to clarify the role of the type of renal replacement therapy on the faecal microbiome.

## Methods

### Patients

We enrolled stable patient with ESRD undergoing HD or PD at the Department of Internal Medicine, Clinical Division of Nephrology, Medical University of Graz, Austria. The Ethics Committee of the Medical University Graz (IRB00002556) approved the study protocols (23-056 ex10/11, NCT01362569). The study was conducted according to the Declaration of Helsinki and all participants gave written informed consent prior enrolment. Patients with malignancy, pregnancy, chronic inflammatory bowel disease, celiac disease, active alcohol abuse, severe organ dysfunction unrelated to renal dysfunction or patients with clinical evidence of active infection were excluded. We obtained routine biochemistry data midweek immediately before HD or at the monthly outpatient visit in PD patients respectively. Clinical data and data on comorbidities and medication were obtained from the electronic patient charts. For co-medication we assessed if patients took proton pump inhibitors, immunosuppression or phosphate binders at the time of stool sampling. Due to the small sample size and since 7 out of the 30 patients took more than one phosphate binder (sevelamer and either lanthanum-carbonate or calcium-acetate), it was statistically not feasible to test for all of combinations of phosphate binders, therefore we only tested whether sevelamer was used or not. Age and sex matched healthy controls were included in the study. These subjects had no evidence of renal disease.

### Microbiome Analyses

Faecal samples of patients were frozen and stored at −80 °C immediately after they have been received in the study centre in Graz. Patients received written instructions how to collect the stool sample and were advised to transport the sample to the study centre immediately after collection. DNA extraction from faecal samples were performed by mechanical lysis with a MagnaLyser Instrument (Roche Diagnostics, Mannheim, Germany) and subsequent total bacterial genomic DNA isolation with the MagNA Pure LC DNA Isolation Kit III (bacteria, fungi) in a MagNA Pure LC 2.0 Instrument (Roche Diagnostics) according to the manufacturer’s instructions. For amplification of bacterial 16 S rRNA gene the template-specific sequence 27F-AGAGTTTGATCCTGGCTCAG; R357-CTGCTGCCTYCCGTA targeting the hypervariable region V1-2 of the 16 S rRNA gene were used. PCR reactions for each sample were performed in triplicates. Subsequently the amplicons were purified according to standard procedures, quantified, pooled and sequenced with the MiSeq Reagent Kits v3 (600 cycles, Illumina, Eindhoven, Netherlands) according to manufacturer’s instructions with 20% OhiX (Illumina). The generated FASTQ were prepared for analysis using Qiime tools^50^ implemented in Galaxy (www.galaxy.medunigraz.at). Paired end reads were joined by fastq-join tool. Primer was removed by cutadapt 1.6 and USEARCH 6.1^51^ was used for reference (SILVA v123) based chimera detection. Open reference operational taxonomic unit (OTU) picking was done to define present OTUs. Clustering was performed by UCLUST^52^ with a 97% sequence similarity threshold. Fasttree was used to generate a phylogenetic tree. Taxonomic assignment was done using SILVA database^53^, when necessary sequences were blasted in NCBI database for further classification^54^. After performing a quality-filtering based on OTU abundance with removal of OTUs with less than 0.005% sequences of the total number of reads and OTUs that were only present in 1 sample, 10 030 949 pyrosequencing reads with a fraction of non-zero values of 0.061 were obtained. The average count per sample was 147 452 with a range from 87769 to 320731 (Standard deviation 80288). Data was rarefied to 87668 reads.

### Statistics

For statistical comparisons of alpha-diversity metrics (Chao1, observed species, phylogenetic diversity whole tree) a nonparametric t-test with 999 Monte-Carlo permutations was performed and the Bonferroni method was used for multiple comparison corrections. Calculated beta diversity metrics (Bray Curtis, unweighted and weighted UniFrac) were compared by using the nonparametric ANOSIM measure. Significant differences in relative abundances of taxa were calculated by using a nonparametric Kruskal-Wallis test in QIIME 1.9.1 using Benjamini-Hochberg False Discovery Rate (FDR) correction. P-values below 0.05 were considered statistically significant.

The web-based program Calypso 7.14 (http://cgenome.net/calypso/) was used for redundancy analysis (RDA) and Linear discriminative analysis effect size (LEfSe) calculations as well as the visualisation of the obtained results. PICRUSt^55^ implemented in a Galaxy was used to predict Kyoto Encyclopedia of Genes and Genomes (KEGG) functional pathway abundance content from 16 s rDNA data using a closed reference OTU table created in QIIME and the Greengenes reference database–version 13 8^56^.

All other statistical analyses were performed using SPSS version 23.0 (SPSS Inc., Chicago, Illinois, USA) and GraphPad Prism version 7.00 for Windows (GraphPad Software, California, USA) for visualisations. Comparison of two independent groups with normally distributed parameters was tested for homogeneity (Levene’s test) and accordingly assessed by Student’s or Welch’s t-test; three or more groups were compared by ANOVA using Tukey’s or Games-Howell’s correction. For parameters, which were not normally distributed, non-parametric tests were used (Mann Whitney U test, or Kruskal-Wallis). Two-tailed Pearson test was used for metrical data correlation. All statistical tests were 2-sided, and p-values < 0.05 were considered statistically significant. Data are presented as median and quartiles (Q_1_, Q_3_) unless stated otherwise. The sequencing data are available from the European Nucleotide Archive (accession number PRJEB21167).
