# Author Correction: Structural and functional differences in gut microbiome composition in patients undergoing haemodialysis or peritoneal dialysis

**DOI:** 10.1038/s41598-019-43263-x

**Published:** 2019-06-06

**Authors:** Vanessa Stadlbauer, Angela Horvath, Werner Ribitsch, Bianca Schmerböck, Gernot Schilcher, Sandra Lemesch, Philipp Stiegler, Alexander R. Rosenkranz, Peter Fickert, Bettina Leber

**Affiliations:** 1 Division of Gastroenterology and Hepatology, Department of Internal Medicine, Medical University of Graz, Graz, Austria; 2 Division of Transplantation Surgery, Department of Surgery, Medical University of Graz, Graz, Austria; 3 Clinical Division of Nephrology, Department of Internal Medicine, Medical University of Graz, Graz, Austria; 4 Intensive Care Unit, Department of Internal Medicine, Medical University of Graz, Graz, Austria; 5 Center of Biomarker Research in Medicine (CBmed), Graz, Austria

Correction to: *Scientific Reports* 10.1038/s41598-017-15650-9, published online 15 November 2017

This Article contains an error in the Methods section under the subheading ‘Statistics’.

“The sequencing data are available from the European Nucleotide Archive (accession number PRJEB21167).”

should read:

“The sequencing data are available from the NCBI Sequence Read Archive (accession number PRJNA390475).”

